# Role of multisegmental nerve ultrasound in the diagnosis of leprosy neuropathy

**DOI:** 10.1371/journal.pone.0305808

**Published:** 2024-07-18

**Authors:** Andrea De Martino Luppi, Guilherme Emílio Ferreira, Isabella Sabião Borges, Douglas Eulálio Antunes, Lúcio Araújo, Diogo Fernandes dos Santos, Marcello Henrique Nogueira-Barbosa, Isabela Maria Bernardes Goulart

**Affiliations:** 1 National Reference Center for Sanitary Dermatology and Leprosy, Clinics’ Hospital, School of Medicine, Federal University of Uberlândia (UFU), Uberlândia, MG, Brazil; 2 Radiology Division, Clinics’ Hospital, School of Medicine, Federal University of Uberlândia (UFU), Uberlândia, MG, Brazil; 3 Postgraduate Program in Health Sciences, School of Medicine, Federal University of Uberlândia (UFU), Uberlândia, MG, Brazil; 4 Department of Mathematics, Federal University of Uberlândia (UFU), Uberlândia, MG, Brazil; 5 Radiology Division, Department of Medical Imaging, Hematology and Clinical Oncology, Ribeirão Preto Medical School, University of São Paulo Ribeirão Preto, São Paulo, Brazil; BG Trauma Center Ludwigshafen, GERMANY

## Abstract

**Introduction/Aims:**

Leprosy is the most common treatable peripheral neuropathy worldwide. The detection of peripheral nerve impairment is essential for its diagnosis and treatment, in order to prevent stigmatizing deformities and disabilities. This study was performed to identify neural thickening through multisegmental ultrasound (US).

**Methods:**

We assessed US measurements of cross-sectional areas (CSAs) of ulnar, median and tibial nerves at two points (in the osteofibrous tunnel and proximal to the tunnel), and also of the common fibular nerve at the fibular head level in 53 leprosy patients (LP), and compared with those of 53 healthy volunteers (HV), as well as among different clinical forms of leprosy.

**Results:**

US evaluation detected neural thickening in 71.1% (38/53) of LP and a mean number of 3.6 enlarged nerves per patient. The ulnar and tibial were the most frequently affected nerves. All nerves showed significantly higher measurements in LP compared with HV, and also greater asymmetry, with significantly higher values for ulnar and tibial nerves. We found significant CSAs differences between tunnel and pre-tunnel points for ulnar and tibial nerves, with maximum values proximal to the tunnel. All clinical forms of leprosy evaluated showed neural enlargement through US.

**Discussion:**

Our findings support the role of multisegmental US as a useful method for diagnosing leprosy neuropathy, revealing that asymmetry, regional and non-uniform thickening are characteristics of the disease. Furthermore, we observed that neural involvement is common in different clinical forms of leprosy, reinforcing the importance of including US evaluation of peripheral nerves in the investigation of all leprosy patients.

## Introduction

Leprosy is a chronic infectious disease caused by *Mycobacterium leprae* that mainly involves the nerves and skin. Its clinical forms are defined by the host immune response and bacillary load, resulting in a wide clinical spectrum [1, 2]. Leprosy neuropathy is considered the most common peripheral neuropathy of infectious etiology worldwide, representing a public health problem, primarily due to its incapacitating potential by means of functional impairments and deformities, leading to social discrimination and stigma [3, 4]. Therefore, some authors advocate that leprosy should be regarded as a chronic neurological condition rather than a skin disease [5, 6].

Disability in leprosy is a direct consequence of damage to the peripheral nervous system, which may result in autonomic, sensory and motor dysfunction. Neurological involvement can begin before diagnosis, during or after treatment, and can include nerve trunks as well as distal cutaneous branches. Mononeuropathy, multiple mononeuropathy and confluent mononeuropathy are the most common clinical presentations. Sensory symptoms often correspond to the initial and most common complaints [7–10].

Leprosy is classified into five clinical forms, as proposed in 1966 by Ridley-Jopling [11], based on skin lesion histopathology and bacterial load. According to this classification, patients are grouped into two polar forms: cases with cellular immune response mediated by T lymphocytes that are classified as tuberculoid, and cases of anergic patients with humoral response that are considered lepromatous leprosy. Patients between these two extremes are defined as borderline, presenting intermediate immune responses, depending on alterations in the level of immunity and the number of bacteria over time. For operational purposes aiming to achieve proper treatment regimens, patients are divided into paucibacillary (PB) or multibacillary (MB) forms, according to their bacilloscopic index, the number of skin lesions and affected nerves [12, 13].

Currently, nerve assessment in leprosy relies mainly on clinical palpation and on nerve conduction studies, but these techniques have limitations and may therefore lead to treatment delay. Although neurophysiology gives detailed information about dysfunction of affected nerves, it does not show anatomic changes, such as thickening and fascicular pattern changes. High-resolution ultrasound (US) has emerged as an additional and accessible modality by which the morphology of peripheral nerves can be evaluated, identifying anatomical abnormalities over a greater length of the nerve in a few minutes [14–18]. Besides, it is reported that US is more accurate than clinical palpation for evaluation of peripheral nerve enlargement, enabling objective measurements of peripheral nerve thickening and asymmetry [19–22].

This study aimed to investigate neural thickening in leprosy patients by multisegmental US assessment of peripheral nerves, examining differences in US measurements between leprosy patients and healthy volunteers, and also among different clinical forms of leprosy according to the Ridley-Jopling classification.

## Materials and methods

### Ethics statement

We recruited leprosy patients from the National Reference Center of Sanitary Dermatology and Leprosy in Brazil, under the approval of the Ethics Committee of the Federal University of Uberlândia (CAAE: 23136419.3.0000.5152). Written informed consent was given by all adult participants, and was obtained from parents of participating minors on their behalf.

### Type of study and subjects

This is a cross-sectional study composed of 2 groups, encompassing 53 leprosy patients (LP) and 53 healthy volunteers (HV). The individuals of the LP group were enrolled by intentional sampling at the National Reference Center for Sanitary Dermatology and Leprosy–Clinical Hospital, Medical School, Federal University of Uberlândia, attended from May to June 2021.

As eligibility criteria, for the LP group, participants should have a diagnosis of leprosy according to the criteria of the World Health Organization (WHO) and not yet have completed the fixed-duration treatment were included in the study. The group of HVs was composed of participants of the same population from an endemic region for leprosy, but without a diagnosis of this disease neither a history of household contact with leprosy cases. They underwent multisegmental US evaluation of peripheral nerves by the same investigator who performed the leprosy patient exams.

Participants who presented other possible etiologies of peripheral neuropathies, such as diabetes mellitus, hypothyroidism, hepatitis B or C, human immunodeficiency virus infection, hereditary neuropathies or chronic alcoholism were excluded from both groups.

### Clinical characterization

Leprosy diagnosis was established based on clinical signs and symptoms through a rigorous dermato-neurological evaluation, bacilloscopy, serological and molecular tests, routinely performed at this center.

During dermato-neurological examination, each LP was carefully inspected not only for skin lesions, but also for neurological impairment, through the presence of sensory and motor impairment. All LPs underwent a rigorous sensory evaluation, ruling out the impairment of all sensory modalities (pain, temperature, touch and vibration). Furthermore, the LP group underwent clinical palpation of nerves, always by the same trained professional, to detect peripheral nerve thickening: ulnar nerve at the elbow, common fibular at the fibular head and tibial nerve at the ankle. Clinical palpation of the median nerve could not be performed due to its deeper location. It is noteworthy that all HVs were submitted to dermato-neurological examination to rule out leprosy, even in the absence of epidemiological antecedents.

The level of functional disability was assessed by expert professionals in all LPs according to the recommended protocol of the Ministry of Health [23], which evaluates the neural functional integrity and the degree of physical disability, through muscle strength and sensory tests of the hands and feet. Disability grade 2 was established based on the presence of visible deficiencies, such as claws (clawing of digits), bone resorption, muscular atrophy, contractures and wounds.

In summary, epidemiological characteristics (age, gender) and clinical variables (clinical form according to the Ridley-Jopling classification, degree of disability, presence of enlarged nerve on US exam) were evaluated in this study.

### Laboratory analyzes

All LP underwent the following laboratory tests, as described below.

Identification of acid-fast bacilli (AFB)–This analysis was performed on slit skin smears from six sites (two ear lobes, two elbows, two knees), as well as on skin biopsy samples. Sample collection was preceded by topical application of cream containing lidocaine (7%) and tetracaine (7%) at all sites.

ELISA anti-PGL-I IgM serology–Serum anti-PGL-I IgM antibodies were detected by ELISA performed against the purified native PGL-I from the *M*. *leprae* cell wall. The reagent was obtained through BEI Resources, NIAID, NIH: Monoclonal Anti-*Mycobacterium leprae* PGL-I, Clone CS-48 (produced in vitro), NR-19370. The titration of anti-PGL-I antibodies was expressed as an ELISA index according to the proportion between the bacillary load of the sample in relation to the cutoff point. Values above 1.0 were considered positive [24].

DNA Extraction and Real Time Quantitative Polymerase Chain Reaction (qPCR) of the following samples: 1- slit-skin smear (one sample) from six sites (two ear lobes, two elbows, two knees); 2- elbow skin biopsy. The qPCR assay to detect *M*. *leprae* DNA was performed by targeting the bacillus-specific genomic region (RLEP) in a real-time PCR system (ABI 7300, Applied Biosystems, Foster City, CA, USA). The vials were always compared to two negative controls to ensure that the sample was not contaminated [25–27].

Based on the clinical findings and laboratory analyses, LPs were separated according to the Ridley-Jopling classification into four groups [11]: borderline-tuberculoid (BT), borderline-borderline (BB), borderline-lepromatous (BL), and lepromatous (LL). We did not obtain patients from the tuberculoid (TT) group in our study.

### Skin biopsy

All of the LP selected did not present any skin lesion. For this reason, the biopsy of a small elbow skin fragment was performed, considering that it is a cold region with possible intradermal neural impairment and, therefore, a site often altered in leprosy neuropathy. A wedge-shaped incision was made using a scalpel blade, and a fragment of approximately 1 cm along its greatest length that reached the hypodermis was removed. One part of the skin sample was sent to the molecular pathology and biotechnology laboratory and the other part was sent to the pathology laboratory for histopathological evaluation. Fite-Faraco staining was used to investigate *M*. *leprae*.

### Ultrasound

All LPs underwent multisegmental US of the peripheral nerves, performed by a Board-certified radiologist, with experience in peripheral nerve imaging, using a 12–13 MHz linear transducer model LOGIC P6 PRO (GE Medical Systems, Milwaukee, Wisconsin, U.S.A). In order to avoid verification bias, the investigator was blinded to the clinical and laboratory characteristics of the LPs, preventing interference in US outcomes.

Participants were examined in a seated position with the arm in abduction and elbow flexed at 45° for assessment of ulnar and knees flexed at 90° for the common fibular nerves. For median nerve examination, the arms of the study participant were positioned by their respective sides and in supination. The tibial nerve was examined in minimal external rotation of the lower limb. Positioning of limbs of study participants during US was kept uniform throughout the study [22, 28].

US measurements were performed at compression sites often affected by leprosy neuropathy. The ulnar nerve was evaluated at the ulnar sulcus in the cubital tunnel (Ut) and at 3 to 4 cm above the medial epicondyle, proximal to the cubital tunnel (Upt). The median nerve was scanned at the wrist in the carpal tunnel (Mt) and 3 to 4 cm above the carpal tunnel (Mpt). The common fibular (CF) nerve was evaluated from at the level of the fibular head. The tibial nerve was scanned at the ankle in the tarsal tunnel (Tt), behind the medial malleolus, and at 3 to 4 cm above the medial malleolus, proximal to the tarsal tunnel (Tpt) [21, 22, 28].

For measuring cross-sectional areas (CSAs) of the nerves, the US beam was kept perpendicular to the nerve to minimize anisotropy. CSAs were measured by freehand delimitation at the inner borders of the echogenic rims of the nerves, using the electronic cursor at the time of examination [22, 28].

CSA measurements were used to determine the CSA index (ΔCSA), which was calculated as the absolute difference between CSAs for each nerve point from one side to the contralateral side. High ΔCSA values reflect nerve asymmetry with the contralateral nerve [22, 29–31].

We also calculated the absolute difference between CSAs measurements of each nerve at the tunnel and proximal to the tunnel points (Δtpt): Mt-Mpt index (ΔMtpt) of the median nerves, Ut-Upt index (ΔUtpt) of the ulnar nerves, and Tt-Tpt index (ΔTtpt) of the tibial nerves. High ΔMtpt, ΔUtpt and ΔTtpt values reflect non-uniform enlargement of the nerves [22, 29, 30].

In summary, as outcome factors, we assessed the following variables: CSA and ΔCSA of each peripheral nerve, and also ΔMtpt, ΔUtpt and ΔTtpt.

As a comparative (control) sample of healthy Brazilian volunteers, we used the database of the radiology laboratory of our service, established by Luppi et al. [32]. Considering that the US assessment of peripheral nerves is still a recent technique, we believe that internal standardization is important. Our sample consisted of 53 individuals, assessed in detail by a radiologist with experience in the evaluation of peripheral nerves. Although there was no pairing specifically for this study, the data obtained in this group are in accordance with the normality standards used in other studies [28, 30, 33].

For the classification of the values of CSA, ΔCSA, ΔMtpt, ΔUtpt and ΔTtpt as normal or abnormal, the measurements obtained in the evaluation of the nerves of HV were used, considering any values greater than mean plus 3 standard deviations as abnormal.

### Statistical analysis

The Shapiro Wilk test was employed to test data normality within groups. As all ultrasound variables did not present normal probability distribution, we performed the Mann-Whitney test to analyze differences between the means of two groups (LPs and HVs) and the Kruskal-Wallis test to verify differences among the four clinical forms of leprosy. The Chi-square test was applied for the study of dichotomous variables. For continuous variables, the Mann-Whitney u test was used. Probability (*p*) values less than 0.05 were considered significant. The procedures were performed using the software Statistical Package for Social Sciences—SPSS Version 20 (IBM, Armonk, NY, USA) for Windows.

## Results

In the group of LPs, there were 32 men (60.4%) and 21 women (39.6%), with a mean age of 46.9 ± 15.3 years, while the group of HVs was composed of 33 women (62.3%) and 20 men (37.7%), with a mean age of 40.9 ± 12.0 years. The clinical data and laboratory characteristics of LPs are presented in Table 1. For clinical data and CSA values of each participant see supporting information (S1 and S2 Tables).

**Table 1:** Clinical and laboratory characteristics among leprosy patients.

Parameters	Leprosy patients (n = 53)
**Ridley-Jopling classification**	
Lepromatous (LL)	39.6% (21/53)
Borderline-Lepromatous (BL)	13.2% (7/53)
Borderline-Borderline (BB)	17.0% (9/53)
Borderline-Tuberculoid (BT)	30.2% (16/53)
**Degree of disability**	
Grade 0	39.6% (21/53)
Grade 1	34.0% (18/53)
Grade 2	26.4% (14/53)
**ELISA index (anti-PGL-1 IgM)**	1.7 ± 1.3 (mean ± SD)
**ELISA anti-PGL-1 IgM serology**	
Negative	34.0% (18/53)
Positive	66.0% (35/53)
**Positivity skin biopsy (Fite-Faraco)**	54.7% (29/53)
**Positivity skin biopsy qPCR**	67.9% (36/53)
**Positivity slit skin smear**	47.1% (25/53)
**Positivity slit skin smear qPCR**	58.5% (31/53)
**Total of patients with enlarged nerves on US exam**	71.7% (38/53)

n: number of leprosy patients; LL: Lepromatous; BL: Borderline-Lepromatous; BB: Borderline-Borderline; BT: Borderline-Tuberculoid; ELISA: enzyme-linked immunosorbent assay; anti-PGL-1 IgM: anti-phenolic glycolipid-1 IgM antibodies; SD: standard deviation; qPCR: Real Time Quantitative Polymerase Chain Reaction; US: Ultrasound.

A total of 424 nerves (one hundred and six nerves each of ulnar, median, common fibular and tibial nerves) were assessed in 53 LPs. We excluded two measurements of the Mt nerve due to suspicion of carpal tunnel syndrome since each of the two measurements corresponded to more than twice the measurement of the same nerve at the pre-tunnel level [34].

**Fig 1:**
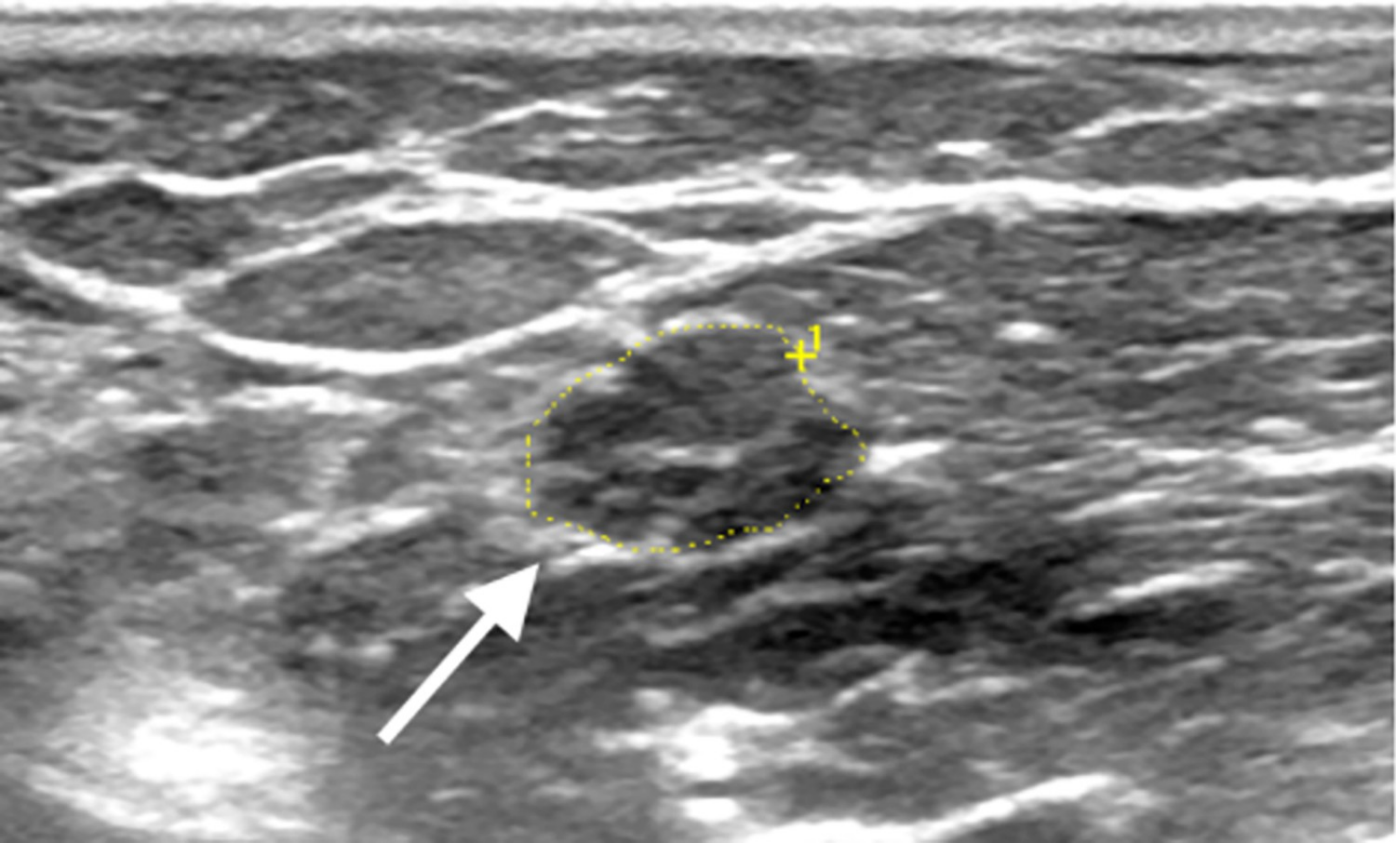
Ultrasound of the ulnar nerve (transverse view) proximal to the cubital tunnel in a leprosy patient, showing neural thickening and enlarged fascicles (white arrow).

**Fig 2:**
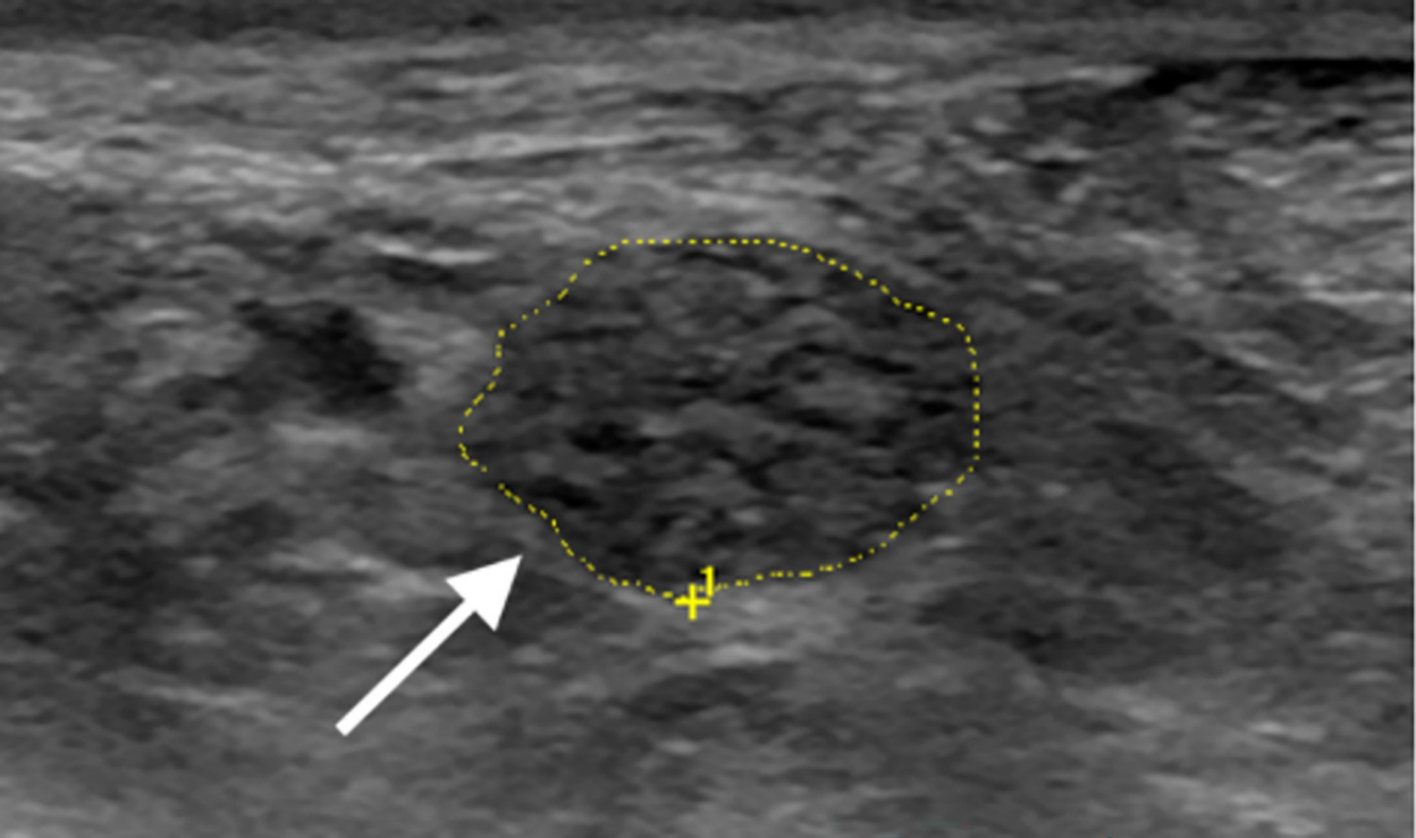
Ultrasound of the tibial nerve (transverse view) proximal to the tarsal tunnel in a leprosy patient, showing a thickened and hypoechoic nerve with loss of the fascicular pattern (white arrow).

Multisegmental US evaluation detected a total of 138 enlarged nerves, implying neural impairment in 71.7% (38/53) of LPs, and a mean number of 3.6 enlarged nerves per patient. US assessment detected only one thickened nerve per patient (mononeuropathy) in 23.7% (9/38), and two or more affected nerves (multiple mononeuropathy) in 76.3% (29/53) of LPs. The most frequently affected nerves by US assessment were the ulnar (Fig 1) and tibial (Fig 2), followed by the median and common fibular, as described in Table 2.

**Table 2:** Distribution of peripheral nerves most involved in multisegmental US evaluation of leprosy patients.

Enlarged nerves	n	%
**Ulnar**	54	39.1
**Tibial**	35	25.4
**Median**	25	18.1
**Common fibular**	24	17.4
**Total of affected nerves**	138	100%

n: number of nerves; %: percentages of nerves.

The following tables show the measures for CSA, ΔCSA, and ΔMtpt, ΔUtpt and ΔTtpt values of the nerves studied in LPs and HVs.

The mean values of CSA of all nerves evaluated were significantly higher in LPs, as described in Table 3. The percentage of enlarged Upt nerves was significantly greater in the group of LPs (p = 0.0024).

**Table 3:** Cross-sectional area (CSA) measurements (mm^2^) by multisegmental ultrasound evaluation in healthy volunteers and leprosy patients.

Peripheral nerves		Healthy volunteers	Leprosy patients	*p* value
**Ulnar (Upt)**	n	106	106	
	Mean ± SD	4.81 ± 1.09	11.3 ± 10.62	0.0000*
	95% CI	[4.6–5.02]	[9.24–13.31]	
	Abnormal (>8.08)	0	47.2% (50/106)	0.0000*
**Ulnar (Ut)**	N	106	106	
	Mean ± SD	5.81 ± 1.24	9.68 ± 5.7	0.0000*
	95% CI	[5.57–6.05]	[8.58–10.78]	
	Abnormal (>9.53)	0	33.0% (35/106)	0.0646
**Median (Mpt)**	N	106	106	
	Mean ± SD	5.97 ± 1.12	9.18 ± 5.78	0.0000*
	95% CI	[5.76–6.19]	[8.08–10.32]	
	Abnormal (>9.33)	0	23.6% (25/106)	0.2169
**Median (Mt)**	n	106	104	
	Mean ± SD	7.62 ± 1.35	9.53 ± 3.48	0.0000*
	95% CI	[7.36–7.88]	[8.74–10.14]	
	Abnormal (>11.67)	0	12.5% (13/104)	0.5667
**CF**	n	106	106	
	Mean ± SD	12.08 ± 2.15	16.68 ± 8.55	0.0000*
	95% CI	[11.66–12.49]	[15.07–18.35]	
	Abnormal (>18.53)	0	22.6% (24/106)	0.2395
**Tibial (Tpt)**	n	106	106	
	Mean ± SD	8.47 ± 1.19	14.66 ± 9.17	0.0000*
	95% CI	[8.24–8.7]	[12.89–16.45]	
	Abnormal (>12.04)	0	32.1% (34/106)	0.0744
**Tibial (Tt)**	n	106	106	
	Mean ± SD	8.47 ± 1.26	13.72 ± 7.63	0.0000*
	95% CI	[8.23–8.71]	[12.24–15.18]	
	Abnormal (>12.25)	0	29.2% (31/106)	0.1105

Upt: ulnar nerve proximal to the cubital tunnel; Ut: ulnar nerve in the cubital tunnel; Mpt: median nerve proximal to the carpal tunnel; Mt: median nerve in the carpal tunnel; CF: common fibular nerve; Tpt: tibial nerve proximal to the tarsal tunnel; Tt: tibial nerve in the tarsal tunnel; n: number of nerves; SD: standard deviation; 95% CI: 95% confidence interval.

*Statistically significant.

We also observed higher ΔCSA measurements for all studied nerves in LPs (Table 4), with significantly higher values for the ulnar and tibial nerves compared to HVs, both in the tunnel and proximal to the tunnel points.

**Table 4:** Absolute difference in CSA (ΔCSA) measurements (mm^2^) between right and left sides by multisegmental US evaluation in study participants.

Peripheral nerves		Healthy volunteers	Leprosy patients	*p* value
**Ulnar (Upt)**	n	53	53	
	Mean ± SD	0.49 ± 0.58	3.63 ± 4.88	0.0000*
	95% CI	[0.33–0.65]	[2.2–4.94]	
	Abnormal (>2.23)	0	37.7% (20/53)	0.1602
**Ulnar (Ut)**	N	53	53	
	Mean ± SD	0.72 ± 0.69	3.24 ± 4.77	0.0033*
	95% CI	[0.53–0.91]	[1.87–4.51]	
	Abnormal (>2.79)	0	35.8% (19/53)	0.1702
**Median (Mpt)**	n	53	52	
	Mean ± SD	0.66 ± 0.71	1.9 ± 5.16	0.0571
	95% CI	[0.47–0.85]	[0.49–3.32]	
	Abnormal (>2.79)	0	9.4% (5/53)	0.8282
**Median (Mt)**	n	53	53	
	Mean ± SD	0.72 ± 0.79	1.36 ± 1.53	0.0743
	95% CI	[0.5–0.94]	[0.89–1.8]	
	Abnormal (>3.09)	0	5.8% (3/52)	0.9425
**CF**	n	53	53	
	Mean ± SD	0.87 ± 0.68	3.12 ± 5.48	0.3242
	95% CI	[0.68–1.06]	[1.55–4.63]	
	Abnormal (>2.91)	0	26.41% (14/53)	0.3535
**Tibial (Tpt)**	n	53	53	
	Mean ± SD	0.83 ± 0.64	3.08 ± 5.19	0.0023*
	95% CI	[0.65–1.01]	[1.59–4.49]	
	Abnormal (>2.75)	0	28.3% (15/53)	0.3108
**Tibial (Tt)**	n	53	106	
	Mean ± SD	0.94 ± 0.79	2.77 ± 4.17	0.0133*
	95% CI	[0.72–1.16]	[1.58–3.89]	
	Abnormal (>3.31)	0	17.0% (9/53)	0.6028

Upt: ulnar nerve proximal to the cubital tunnel; Ut: ulnar nerve in the cubital tunnel; Mpt: median nerve proximal to the carpal tunnel; Mt: median nerve in the carpal tunnel; CF: common fibular nerve; Tpt: tibial nerve proximal to the tarsal tunnel; Tt: tibial nerve in the tarsal tunnel; n: number of nerves; SD: standard deviation; 95% CI: 95% confidence interval.

*Statistically significant.

Moreover, LPs showed greater differences in CSA measurements of ulnar, median and tibial nerves between tunnel points and those proximal to the tunnel (Table 5), and statistically significant differences of ΔUtpt and ΔTtpt values compared to HVs.

**Table 5:** Absolute difference in CSA measurements (mm^2^) between tunnel points and those proximal to the tunnel on the same side by multisegmental US evaluation of study participants.

Peripheral nerves		Healthy volunteers	Leprosy patients	*p* value
**Ulnar (ΔUtpt)**	n	106	106	
	Mean ± SD	1.09 ± 0.88	3.67 ± 8.45	0.0001*
	95% CI	[0.93–1.26]	[2.05–5.33]	
	Abnormal (>3.73)	0	21.7% (23/106)	0.2635
**Median (ΔMtpt)**	n	106	104	
	Mean ± SD	1.71 ± 1.25	2.39 ± 3.45	0.0649
	95% CI	[1.47–1.95]	[1.77–3.11]	
	Abnormal (>5.46)	0	2.9% (3/104)	0.9590
**Tibial (ΔTtpt)**	n	106	106	
	Mean ± SD	0.58 ± 0.60	1.74 ± 3.30	0.0001*
	95% CI	[0.47–0.7]	[1.1–2.38]	
	Abnormal (>2.38)	0	13.21% (14/106)	0.5373

ΔUtpt: Ut-Upt index for ulnar nerve; ΔMtpt: Mt-Mpt index for median nerve; ΔTtpt: Tt-Tpt index for tibial nerve; SD: standard deviation; 95% CI: 95% confidence interval.

*Statistically significant.

Although the mean values of CSAs varied among clinical forms of leprosy according to the Ridley-Jopling classification (Table 6), a statistically significant difference was observed in relation to the CSA measurements of the Upt, Ut ant Tpt nerves. The LL patients showed higher mean values of Upt and Upt CSAs and the highest percentages of thickened ulnar nerves, reaching enlarged Upt and Ut nerve incidences of 61.9% and 42.9%, respectively. We also observed greater Tpt values in the BL group, as well as a higher percentage of abnormal nerves. Furthermore, the percentages of affected Tt nerves were significantly higher between the BL and BB groups. The highest percentages of affected CF nerves were observed between LL and BB. The median nerve did not differ significantly between the groups.

**Table 6:** CSA measurements by multisegmental US evaluation in leprosy patients according to the Ridley-Jopling classification.

Nerve		LL	BL	BB	BT	*p* value
		21 patients	7 patients	9 patients	16 patients	
**Ulnar (Upt)**	n	42	14	18	32	
	Mean ± SD	14.14 ± 14.19	10.24 ± 5.19	10.22 ± 9.41	8.56 ± 5.84	0.0198*
	95% CI	[9.68–18.57]	[7.27–13.21]	[5.56–14.97]	[6.50–10.65]	
	Abnormal	61.9% (26/42)	57.14% (8/14)	38.88% (7/18)	34.37% (11/32)	0.0841
**Ulnar (Ut)**	n	42	14	18	32	
	Mean ± SD	11.03 ± 6.56	9.31 ± 3.02	9.45 ± 6.38	8.21 ± 4.72	0.0397*
	95% CI	[8.99–13.07]	[7.61–11.04]	[6.3–12.65]	[6.47–9.88]	
	Abnormal	42.9% (18/42)	28.57% (4/14)	33.33% (6/18)	21.87% (7/32)	0.2885
**Median (Mpt)**	n	42	14	18	32	
	Mean ± SD	9.26 ± 4.76	10.28 ± 9.92	8.37 ± 4.18	9.07 ± 5.59	0.4480
	95% CI	[7.79–10.77]	[4.51–15.98]	[6.27–10.55]	[7.05–11.11]	
	Abnormal	21.43% (9/42)	28.57% (4/14)	33.33% (6/18)	18.75% (6/32)	0.6446
**Median (Mt)**	n	40	14	18	32	
	Mean ± SD	9.58 ± 3.63	8.61 ± 1.85	9.64 ± 3.05	9.81 ± 4.08	0.9220
	95% CI	[8.09–10.55]	[7.58–9.65]	[8.12–11.12]	[8.37–11.33]	
	Abnormal	15.0% (6/40)	7.14% (1/14)	11.11% (2/18)	12.5% (4/32)	0.8901
**CF**	n	42	14	18	32	
	Mean ± SD	17.65 ± 10.76	15.33 ± 3.26	17.94 ± 8.45	15.29 ± 6.77	0.4801
	95% CI	[14.33–21.01]	[13.47–17.2]	[13.72–22.14]	[12.92–17.8]	
	Abnormal	28.57% (12/42)	7.14% (1/14)	33.33% (6/18)	15.62% (5/32)	0.0585
**Tibial (Tpt)**	n	42	14	18	32	
	Mean ± SD	14.44 ± 7.1	18.3 ± 11.74	12.89 ± 5.39	14.36 ± 11.69	0.0352*
	95% CI	[12.21–16.68]	[11.6–25.21]	[10.14–15.56]	[10.12–18.58]	
	Abnormal	25.57% (12/42)	57.14% (8/14)	38.88% (7/18)	21.87% (7/32)	0.1029
**Tibial (Tt)**	n	42	14	18	32	
	Mean ± SD	13.02 ± 4.82	16.91 ± 9.73	13.19 ± 4.97	13.53 ± 10.37	0.0861
	95% CI	[11.52–14.52]	[11.34–22.52]	[10.73–15.70]	[9.76–17.24]	
	Abnormal	21.43% (9/42)	57.14% (8/14)	50% (9/18)	18.75% (6/32)	0.0365*

LL: Lepromatous; BL: Borderline-Lepromatous; BB: Borderline-Borderline; BT: Borderline-Tuberculoid; Upt: ulnar nerve proximal to the cubital tunnel; Ut: ulnar nerve in the cubital tunnel; Mpt: median nerve proximal to the carpal tunnel; Mt: median nerve in the carpal tunnel; CF: common fibular nerve; Tpt: tibial nerve proximal to the tarsal tunnel; Tt: tibial nerve in the tarsal tunnel; n: number of nerves; SD: standard deviation; 95% CI: 95% confidence interval.

*Statistically significant.

## Discussion

The present study systematically evaluated the measurements of the peripheral nerves of upper and lower limbs in LPs assisted in a national reference center for leprosy in Brazil, using a multisegmental US method, and also investigated differences among their clinical forms according to the Ridley-Jopling classification. Several previous studies had already investigated measurements of the nerves in leprosy neuropathy by US [14, 18–22, 31, 35], but this is one of the few studies available in the literature that simultaneously evaluated the median, ulnar and tibial nerves in a multisegmental technique [29, 32, 36].

In recent years, US has been described as a diagnostic tool for diseases of the peripheral nervous system, considered a non-invasive technique, usually painless and more cost-effective than other imaging methods, such as magnetic resonance imaging (MRI) [15–17, 37]. The improvement in image quality and the reduction in the size and cost of the devices will make US an accessible diagnostic tool in leprosy-endemic countries. Moreover, US is able to study a longer segment of nerve than MRI and detect structural changes in nerve sites that cannot be biopsied for histopathology [18, 19]. Previous studies have shown that peripheral nerve thickening is a highly characteristic finding in patients diagnosed with leprosy, which can be objectively measured by the corresponding CSA in the US assessment [14, 18–22]. Additionally, US can detect nerve enlargement in patients diagnosed with leprosy without functional abnormality identified in electroneuromyographic studies. In a study by Elias Jr et al. [21], the US evaluation of three patients showed ulnar nerve thickening without electrophysiological nerve abnormalities, indicating that an enlarged peripheral nerve may present normal function, and corroborating the importance of performing US as a complementary exam to neurophysiological studies during the investigation of these patients, providing important anatomic information [21, 38].

In our study, the prevalence of leprosy neuropathy was 71.7%, considering the 53 cases assessed during this period, and all the nerves studied showed significantly higher measurements in LPs compared with HVs, both in the tunnel and proximal to the tunnel, demonstrating the importance of multisegmental US method in detecting neural enlargement in leprosy [29, 32, 36]. Multisegmental US evaluation detected multiple mononeuropathy in the majority of the cases, corroborating previous studies reporting that MB patients have a greater number of altered nerves [5, 8, 14]. The ulnar nerve was the most commonly involved nerve, consistent with the results of previous analyses, including clinical studies [8–10, 19, 22]. Lugão et al. [22] found that up to 81.3% of LL patients showed abnormal ulnar nerve CSA values in the cubital tunnel level. The tibial nerve was the second most affected in our study, but a less commonly evaluated nerve in prior studies [20, 22, 31], followed by the median and the common fibular nerves. We also found that the ΔCSA for all studied nerves were higher in LPs compared with HVs, with significantly higher values for the ulnar and tibial nerves. Frade et al. [31] had already observed that LPs had greater ΔCSAs than the HVs for the ulnar and the common fibular nerves. Recent studies published by Voltan et al. [29, 36] and Luppi et al. [32] also confirmed the asymmetrical pattern of involvement in leprosy neuropathy detected by US. Therefore, our findings corroborate with previous analyses, which indicate that neural hypertrophy and asymmetry detected at US are characteristics of leprosy neuropathy [22, 29, 31, 32].

Furthermore, we observed greater differences in CSA measurements between the tunnel and proximal to the tunnel of the ulnar, median and tibial nerves in LPs compared to HVs, reflecting regional and non-uniform neural thickening in leprosy neuropathy [29, 36, 37, 38]. There were statistically significant differences in ΔUtpt and ΔTtpt values, with higher measurements observed a few centimeters above the tunnel level. Although the median nerve did not differ statistically between the tunnel and pre-tunnel points, thickened nerves were more numerous above the tunnel level [33]. Previous studies have shown that ΔUtpt possess high specificity for diagnosis of leprosy [21, 22, 38]; however, this is one of the first studies in which this finding was described for the tibial nerve. A recent study published by our research group [32] evaluated the peripheral nerves of leprosy househould contacts using the multisegmental US technique and detected neural thickening in 26.5% (13/49) of seropositive contacts, with significantly higher CSA values of the common fibular and tibial nerves and also significantly greater asymmetry in the common fibular and tibial nerves (proximal to the tunnel) in this group. Although nerve enlargement can be found in other types of neuropathy, this regional pattern of neural thickening in leprosy, with maximum CSA values proximal to the osteofibrous tunnel, is another feature of leprosy neuropathy [29, 36–38]. Therefore, our results showed the importance of using a multisegmental US technique for the proper diagnosis of this regional and non-uniform characteristic of peripheral nerve enlargement in leprosy patients, helping to discriminate leprosy from other etiologies of neuropathy, especially acquired immune-mediated neuropathies and hereditary hypertrophic neuropathies [37].

Peripheral nerve USG can be an important tool in the management of patients with indication for peripheral neural surgical decompression. It is used as a complementary therapy to clinical neuritis treatment with the objective of preserving neural function [39]. The morphological assessment of the peripheral nerve can be useful in choosing the surgical technique to be used, since as we observed in our results, neural thickening is also present in the segments proximal to the classic compression sites. It is important to highlight that the presence of neural thickening is not always accompanied by a sensory-motor deficit. However, in the presence of minimal deficit, evidence of neural thickening favors early decompression, preventing hypoperfusion of the peripheral nerve.

Regarding the variability of mean CSA values of the peripheral nerves between the different types of leprosy according to the Ridley-Jopling classification, we observed peripheral nerve involvement in all clinical forms evaluated, corroborating that leprosy is a classic hypertrophic neuropathy [2, 10, 14, 29, 37]. Our findings showed a statistically significant difference of CSA mean values of the ulnar nerve (pre-tunnel and at the cubital tunnel) and of the tibial nerve proximal to the tarsal tunnel. The LL group showed the greatest mean CSA values and percentages of abnormal ulnar nerves, whereas the BL group showed the highest mean CSA values and percentages of abnormal tibial nerves, corroborating a previous analysis reported by Lugão et al. [22]. Prior studies showed that thickening of peripheral nerves was also frequent between PB patients and nerve asymmetry detected on US did not significantly differ between PB and MB, demonstrating that enlargement and asymmetry are a characteristic of leprosy neuropathy, regardless of its classification [14, 22].

Although this is one the first studies to evaluate nerve thickening in patients diagnosed with leprosy using the multisegmental US technique, the number of subjects was small, and more studies need to be replicated to confirm our findings, aiming to improve the effectiveness of US in identifying leprosy neuropathy.

In conclusion, we propose the multisegmental US method for evaluation of the peripheral nerves of the upper and lower limbs in the investigation of leprosy neuropathy. Multisegmental US can help to discriminate leprosy from other neuropathy etiologies by revealing that asymmetry, regional and non-uniform hypertrophy, most evident above the osteofibrous tunnel of the nerves, are characteristics of this condition. We observed that neural involvement is common in different clinical forms of the disease, supporting the idea that leprosy is primarily a neurological condition and reinforcing the importance of including multisegmental US evaluation of peripheral nerves in the investigation of all patients diagnosed with leprosy.

## Supporting information

S1 Table Clinical data and CSA measurements of each leprosy patient included in the study. ID: participant identification; RJ: Ridley-Jopling classification; BT: borderline-tuberculoid; BB: borderline-borderline; BL: borderline-lepromatous; LL: lepromatous; Upt: ulnar nerve proximal to the cubital tunnel; Ut: ulnar nerve at the cubital tunnel; Mpt: median nerve proximal to the carpal tunnel; Mt: median nerve at the carpal tunnel; Tpt: tibial nerve proximal to the tarsal tunnel; Tt: tibial nerve at the tarsal tunne; NI: measurement not included (evidence of carpal tunnel syndrome).
(DOCX)

S2 Table Epidemiological data and CSA measurements of each healthy volunteer included in the study. ID: participant identification; Upt: ulnar nerve proximal to the cubital tunnel; Ut: ulnar nerve at the cubital tunnel; Mpt: median nerve proximal to the carpal tunnel; Mt: median nerve at the carpal tunnel; Tpt: tibial nerve proximal to the tarsal tunnel; Tt: tibial nerve at the tarsal tunnel.
(DOCX)

## Abbreviations

Anti-PGL-1 IgM: anti-phenolic glycolipid-1 IgM antibodies
BT: Borderline-tuberculoid
BB: Borderline-borderline
BL: Borderline-lepromatous
CF: common fibular nerve
CREDESH: National Reference Center for Sanitary Dermatology and Leprosy
CSA: cross-sectional area
ELISA: enzyme-linked immunosorbent assay
HV: healthy volunteers
LL: lepromatous
LP: leprosy patients
MB: multibacillary
Mpt: median nerve proximal to the carpal tunnel
MRI: magnetic resonance imaging
Mt: median nerve in the carpal tunnel
p: probability
PB: paucibacillary
qPCR: quantitative real-time PCR
Real-Time PCR: Real-Time Quantitative Polymerase Chain Reaction
SD: standard deviation
Tpt: tibial nerve proximal to the tarsal tunnel
Tt: tibial nerve in the tarsal tunnel
Upt: ulnar nerve proximal to the cubital tunnel
US: ultrasound
Ut: ulnar nerve in the cubital tunnel
WHO: World Health Organization
ΔCSA: CSA index for
ΔMtpt: Mt-Mpt index for median nerve
ΔTtpt: Tt-Tpt index for tibial nerve
ΔUtpt: Ut-Upt index for ulnar nerve
95% CI: 95% confidence interval
