# Supplementary material for: Role of multisegmental nerve ultrasound in the diagnosis of leprosy neuropathy
Source: PLoS One. 2024 Jul 18;19(7):e0305808. doi: 10.1371/journal.pone.0305808 (PMC11257231; doi:10.1371/journal.pone.0305808)
Supplement: S1 Table — ID: participant identification; RJ: Ridley-Jopling classification; BT: borderline-tuberculoid; BB: borderline-borderline; BL: borderline-lepromatous; LL: lepromatous; Upt: ulnar nerve proximal to the cubital tunnel; Ut: ulnar nerve at the cubital tunnel; Mpt: median nerve proximal to the carpal tunnel; Mt: median nerve at the carpal tunnel; Tpt: tibial nerve proximal to the tarsal tunnel; Tt: tibial nerve at the tarsal tunne; NI: measurement not included (evidence of carpal tunnel syndrome). (DOCX) [file pone.0305808.s001.docx]

**Table S1. Clinical data and CSA measurements of each leprosy patient included in the study.**

|  |  |  |  | **CSA measurements (mm²) for each nerve** | | | | | | | | | | | | | |
| --- | --- | --- | --- | --- | --- | --- | --- | --- | --- | --- | --- | --- | --- | --- | --- | --- | --- |
| **ID** | **RJ** | **Gender** | **Age**  **(years)** | **Right Upt** | **Right Ut** | **Right Mpt** | **Right Mt** | **Right common fibular** | **Right Tpt** | **Right Tt** | **Left Upt** | **Left Ut** | **Left Mpt** | **Left Mt** | **Left common fibular** | **Left Tpt** | **Left Tt** |
| 1 | LL | Female | 24 | 11,04 | 10,58 | 5,76 | 7,74 | 8,2 | 12,4 | 11,66 | 5,52 | 7,55 | 7,07 | 8,73 | 9,68 | 11,45 | 11,33 |
| 2 | LL | Male | 42 | 7,63 | 8,72 | 6,83 | 8,66 | 10,15 | 10,9 | 9 | 12,54 | 8,72 | 6,18 | 8,78 | 10,77 | 11,89 | 10,77 |
| 3 | LL | Male | 23 | 9,3 | 7,5 | 6,78 | 9,03 | 11,97 | 13,46 | 12,68 | 18,13 | 20,55 | 6,9 | 8,77 | 10,32 | 11,07 | 11,44 |
| 4 | LL | Male | 24 | 14,51 | 11,37 | 20,6 | 20,77 | 20,54 | 18,62 | 15,76 | 14,13 | 6,85 | 21,28 | 19,8 | 23,69 | 25,15 | 23,72 |
| 5 | BL | Male | 55 | 6,75 | 5,97 | 4,65 | 9,01 | 10,5 | 10,85 | 11,75 | 14,56 | 10 | 6,99 | 8,83 | 10,54 | 10,14 | 10,47 |
| 6 | LL | Male | 59 | 5,48 | 6,96 | 7,49 | 8,31 | 20,28 | 9,72 | 10,49 | 5,43 | 13,8 | 8,88 | 9 | 20,11 | 11,4 | 12,59 |
| 7 | BL | Female | 57 | 4,94 | 7,77 | 6,28 | 7,4 | 15,75 | 9 | 9,75 | 5,81 | 8,02 | 6,17 | 6,97 | 15,45 | 9,37 | 9,11 |
| 8 | LL | Female | 38 | 4,5 | 6 | 5,4 | 6 | 13,8 | 10,6 | 10,1 | 4,6 | 5,6 | 4,8 | 5,9 | 13,2 | 8,8 | 8,4 |
| 9 | BL | Male | 52 | 19,38 | 15,91 | 5,14 | 9,01 | 12,71 | 15,55 | 14,47 | 5,63 | 5,98 | 5,12 | 8,36 | 13,45 | 11,44 | 9,55 |
| 10 | BT | Female | 60 | 4,01 | 4,12 | 6,01 | 6,84 | 12,51 | 8,18 | 8,02 | 3,56 | 3,59 | 5,17 | 6,78 | 12,81 | 7,32 | 7,81 |
| 11 | BB | Male | 39 | 14,2 | 13,71 | 16,55 | 16,3 | 18,34 | 14,96 | 16,14 | 25,94 | 11,85 | 14,96 | 17 | 15,44 | 17,38 | 18,95 |
| 12 | BT | Male | 75 | 4,69 | 4,48 | 9,91 | 6,45 | 10,41 | 10,76 | 11,23 | 5,19 | 5,6 | 11,09 | 13,13 | 10,04 | 10,98 | 10,72 |
| 13 | LL | Female | 26 | 14,37 | 13,63 | 5,78 | 7,22 | 12,78 | 12,29 | 11,56 | 13,18 | 13 | 12,96 | 10,34 | 12,88 | 10,94 | 11,84 |
| 14 | BL | Female | 49 | 10,57 | 11,77 | 5,32 | 5,5 | 16,68 | 14,11 | 14,56 | 9,43 | 9,76 | 5,66 | 6,23 | 15,58 | 8,87 | 9,06 |
| 15 | BT | Female | 78 | 12,76 | 17,82 | 24,58 | 21,65 | 17,1 | 45,65 | 36,05 | 17,64 | 26,69 | 27,2 | 22,71 | 29,35 | 40,28 | 21,49 |
| 16 | LL | Male | 42 | 21,45 | 22,05 | 15,55 | 19,32 | 43,17 | 25 | 23,22 | 19,78 | 18,24 | 16,09 | 15,74 | 32,64 | 28,36 | 25,43 |
| 17 | BL | Male | 44 | 11,75 | 12,43 | 10,58 | 11,19 | 14,54 | 17,77 | 16,95 | 10,81 | 9,05 | 9,75 | 8,64 | 15,25 | 15,85 | 15,91 |
| 18 | LL | Male | 44 | 69,52 | 9,15 | 16,78 | 13,56 | 19,54 | 18,22 | 9,77 | 69,36 | 9,3 | 17,31 | 12,8 | 25,09 | 23,29 | 13,16 |
| 19 | BB | Female | 61 | 3,44 | 12,67 | 5,17 | 8,91 | 17,09 | 24,11 | 22,68 | 3,75 | 6,67 | 5,13 | 9,3 | 26,1 | 23,98 | 20,63 |
| 20 | BB | Female | 47 | 12,74 | 13,2 | 11,62 | 11,34 | 23,94 | 16,03 | 14,91 | 9,03 | 8,27 | 11,66 | 10 | 22,82 | 18,76 | 17,32 |
| 21 | LL | Male | 37 | 15,56 | 7,07 | 6,58 | 6,04 | 17,01 | 32,51 | 30,23 | 11,62 | 6,06 | 5,59 | 6,54 | 12,8 | 18,8 | 19,14 |
| 22 | BB | Male | 63 | 5,2 | 6,38 | 13,63 | 8,95 | 12,28 | 9,24 | 9,2 | 11,28 | 6,54 | 14,46 | 10,85 | 13,25 | 9,26 | 8,94 |
| 23 | BB | Male | 54 | 27,51 | 18,05 | 6,84 | 10,76 | 20,77 | 14,52 | 18,1 | 34,05 | 29,68 | 6,7 | 11,94 | 43,53 | 10,51 | 15,79 |
| 24 | BT | Male | 30 | 18,59 | 8,78 | 5,89 | 9,73 | 9,51 | 17,37 | 16,46 | 20,06 | 8,18 | 6,69 | 6,88 | 9,56 | 15,83 | 15,68 |
| 25 | BT | Male | 41 | 7,52 | 8,39 | 8,64 | 10,72 | 24,6 | 10,4 | 11,39 | 6,33 | 7,58 | 9,31 | 11,75 | 20,27 | 11,04 | 12,31 |
| 26 | BL | Male | 29 | 13,3 | 9,29 | 13,34 | 8,87 | 23,44 | 26,51 | 23 | 20,25 | 6,35 | 16,13 | 12,5 | 16,92 | 22,29 | 19,65 |
| 27 | BL | Female | 59 | 5,24 | 5,62 | 6,16 | 7,95 | 15,24 | 34,74 | 28,28 | 4,87 | 12,4 | 42,58 | 10,08 | 18,54 | 49,66 | 44,28 |
| 28 | LL | Female | 26 | 16,44 | 11,43 | 17,43 | 9,97 | 10,84 | 11,76 | 11,2 | 15,89 | 15,74 | 21,12 | 9,47 | 11,02 | 15,19 | 12,16 |
| 29 | BT | Male | 46 | 5,5 | 6,63 | 6,08 | 6,6 | 12,52 | 7,95 | 7,72 | 5,89 | 6,91 | 6,48 | 7,56 | 12,09 | 7,62 | 7,69 |
| 30 | LL | Male | 32 | 11,47 | 12,95 | 5,82 | 8,99 | 12,91 | 9,34 | 10,21 | 8,6 | 11,52 | 5,92 | 7,33 | 12,49 | 13,94 | 16,08 |
| 31 | LL | Female | 48 | 11,5 | 10,7 | 6,97 | NI | 33,3 | 12,93 | 12,56 | 14,4 | 14,5 | 6,13 | NI | 20,22 | 12,27 | 12,93 |
| 32 | BB | Female | 32 | 4,54 | 5,6 | 5,09 | 7,91 | 12,95 | 6,5 | 7,21 | 5,48 | 5,27 | 6,65 | 7,65 | 12,76 | 6,76 | 7,83 |
| 33 | LL | Female | 63 | 4,57 | 5,68 | 6,92 | 7,04 | 14,61 | 8,64 | 8,82 | 4,42 | 6,46 | 7,2 | 11,28 | 14,56 | 9,8 | 10,08 |
| 34 | BB | Female | 37 | 4,59 | 6,01 | 5,32 | 6,41 | 10,19 | 11,15 | 11,82 | 4,61 | 5,96 | 5,31 | 6,61 | 9,63 | 12,53 | 13 |
| 35 | LL | Male | 38 | 9 | 5,23 | 8,57 | 10,33 | 15,03 | 11,3 | 10,23 | 6,21 | 5,91 | 8,08 | 8,42 | 14,31 | 12,38 | 12,1 |
| 36 | BT | Female | 63 | 5,2 | 6,35 | 4,35 | 6,81 | 18,51 | 14,81 | 12,35 | 4,17 | 6,18 | 4,71 | 6,72 | 18,35 | 8,52 | 10,27 |
| 37 | BT | Female | 48 | 4,8 | 6,84 | 7,85 | 9,49 | 10,24 | 6,99 | 7,86 | 4,76 | 6,04 | 7,07 | 11,21 | 9,79 | 6,77 | 7,49 |
| 38 | LL | Female | 63 | 5,07 | 7,84 | 7,82 | 8,44 | 11,56 | 9,08 | 10,25 | 5,27 | 7,5 | 7,8 | 7,19 | 10,14 | 10,58 | 9,77 |
| 39 | BB | Male | 17 | 4,47 | 4,9 | 6,97 | 7,81 | 11,99 | 8,41 | 7,64 | 4,45 | 4,81 | 6,39 | 7,8 | 12,08 | 9,8 | 9,57 |
| 40 | BT | Male | 57 | 11,9 | 11,4 | 16,2 | 11,77 | 16,9 | 11,96 | 12,31 | 15,79 | 10,57 | 16,08 | 11,71 | 16,03 | 9,83 | 9,38 |
| 41 | BT | Male | 84 | 24,1 | 14,31 | 8,32 | 10,73 | 33,06 | 37,95 | 35,89 | 16,6 | 14,13 | 19,22 | 17,6 | 35,89 | 51,25 | 55,88 |
| 42 | LL | Male | 26 | 5,17 | 5,47 | 7,47 | 7,95 | 9,88 | 9,44 | 9,1 | 12,04 | 9,82 | 9,75 | 10,35 | 10,03 | 9,37 | 9,14 |
| 43 | BT | Female | 33 | 4,42 | 5,57 | 5,07 | 6,52 | 12,72 | 8,27 | 8,24 | 3,95 | 5,8 | 5,55 | 6,7 | 12,47 | 8,72 | 7,72 |
| 44 | BT | Female | 31 | 4,21 | 4,58 | 7,88 | 9,32 | 10,65 | 11,66 | 11,15 | 3,57 | 3,95 | 7,09 | 8,76 | 11,4 | 11,42 | 12,24 |
| 45 | BB | Male | 62 | 4,62 | 5,09 | 4,01 | 6,96 | 12,62 | 8,77 | 8,85 | 4,12 | 5,5 | 4,12 | 7 | 27,17 | 9,32 | 8,88 |
| 46 | BT | Male | 66 | 10,83 | 12,02 | 6,41 | 9,73 | 16,54 | 9,18 | 10,18 | 4,59 | 6,67 | 7,43 | 9,67 | 14,79 | 10,12 | 10,3 |
| 47 | LL | Male | 54 | 5,85 | 6,82 | 6,98 | 5,48 | 38,38 | 12,44 | 12,28 | 4,61 | 5,52 | 9,05 | 8,13 | 64,01 | 12,59 | 12,81 |
| 48 | LL | Male | 55 | 35,6 | 34,9 | 6,12 | 9,26 | 12,11 | 11,78 | 10 | 9,62 | 10,94 | 4,53 | 8,82 | 11,93 | 10,6 | 10,76 |
| 49 | BT | Male | 62 | 4,76 | 5,42 | 5,82 | 7,68 | 12,31 | 9,26 | 7,93 | 4,3 | 4,45 | 5,52 | 6,62 | 9,27 | 9,39 | 8,14 |
| 50 | BT | Male | 43 | 8,09 | 8,97 | 7,87 | 8,23 | 11,64 | 9,16 | 8,9 | 9 | 8,25 | 8,7 | 8,25 | 10,63 | 9,09 | 10,16 |
| 51 | BT | Female | 60 | 4,88 | 6,32 | 6,71 | 8,48 | 13,36 | 9,95 | 9,53 | 12,72 | 6,12 | 5,26 | 7,23 | 13,81 | 11,92 | 10,6 |
| 52 | LL | Male | 41 | 29,85 | 32,33 | 6,03 | 6,55 | 13,68 | 12,71 | 11,31 | 14,06 | 14,48 | 9,04 | 9,34 | 14,63 | 11,83 | 11,85 |
| 53 | LL | Male | 47 | 6,39 | 7,43 | 8,09 | 8,25 | 18,36 | 42,65 | 18,79 | 10 | 7,3 | 7,26 | 7,38 | 18,66 | 11,04 | 12,2 |

Legend: ID: participant identification; RJ: Ridley-Jopling classification; BT: borderline-tuberculoid; BB: borderline-borderline; BL: borderline-lepromatous; LL: lepromatous; Upt: ulnar nerve proximal to the cubital tunnel; Ut: ulnar nerve at the cubital tunnel; Mpt: median nerve proximal to the carpal tunnel; Mt: median nerve at the carpal tunnel; Tpt: tibial nerve proximal to the tarsal tunnel; Tt: tibial nerve at the tarsal tunne; NI: measurement not included (evidence of carpal tunnel syndrome).
